# Implementing robotics and artificial intelligence

**DOI:** 10.7554/eLife.80609

**Published:** 2022-07-20

**Authors:** Sujith Sebastian

**Affiliations:** 1 Clinical Biotechnology Centre, Cellular and Molecular Therapies, NHS Blood and Transplant Bristol United Kingdom

**Keywords:** laboratory automation, labdroid, bayesian optimization, regenerative medicine, ips cell, retinal pigment epithelium, Human

## Abstract

An automated platform for cell culture combines robotics and artificial intelligence to optimize cell culture protocols and reliably produce specific cell types that could be used for regenerative medicine treatments.

**Related research article** Kanda GN, Tsuzuki T, Terada M, Sakai N, Motozawa N, Masuda T, Nishida M, Watanabe CT, Higashi T, Horiguchi SA, Kudo T, Kamei M, Sunagawa GA, Matsukuma K, Sakurada T, Ozawa Y, Takahashi M, Takahashi K, Natsume T. 2022. Robotic search for optimal cell culture in regenerative medicine. *eLife* **11**:e77007. doi: 10.7554/elife.77007.

Most animals, including humans, are made up of different organs and tissues formed by specialized cells that perform specific roles. When tissues degenerate or become damaged, the affected cells have to be replaced so the tissues can keep performing their roles. This regenerative potential exists thanks to populations of stem cells in each tissue, which can divide to produce more stem cells – maintaining a constant pool of stem cells for repair – or differentiate into specialized cells to replace damaged cells.

The division and differentiation of stem cells needs to be in balance: if too many stem cells differentiate, the pool of stem cells could become exhausted, but if the stem cells divide uncontrollably this could lead to cancer. However, this balance often fails with age, or due to environmental or genetic reasons. One of the goals of regenerative medicine is to produce differentiated cells in the laboratory that can be used to heal tissues when they have lost the ability to regenerate for themselves (Pizevska et al., 2022). These treatments could have many potential long-term health benefits, including extending life expectancy, and research into these treatments is increasing both in the laboratory and in the clinic.

In order to use cells differentiated in the laboratory in a clinical setting, it is essential that the protocols used to produce the desired cell types reproducibly, and in high enough numbers. This is difficult to do because differentiating cells are highly sensitive to stimuli in their environments, meaning that their culture conditions have to be carefully controlled (Figure 1). Human operators introduce an added layer of variability that is difficult to control for, since each person does cell culture a little differently, and the protocols used to manufacture regenerative medicine products are often complex (Sebastian et al., 2019). Additionally, the requirements that cells and cell-derived products need to meet to be used in the clinic are changing rapidly, and the tests used to assess these requirements can also propagate variability (Simon et al., 2016). As a result of these issues, the quality of the cells produced for regenerative medicine treatments is often unreliable.

**Figure 1.**
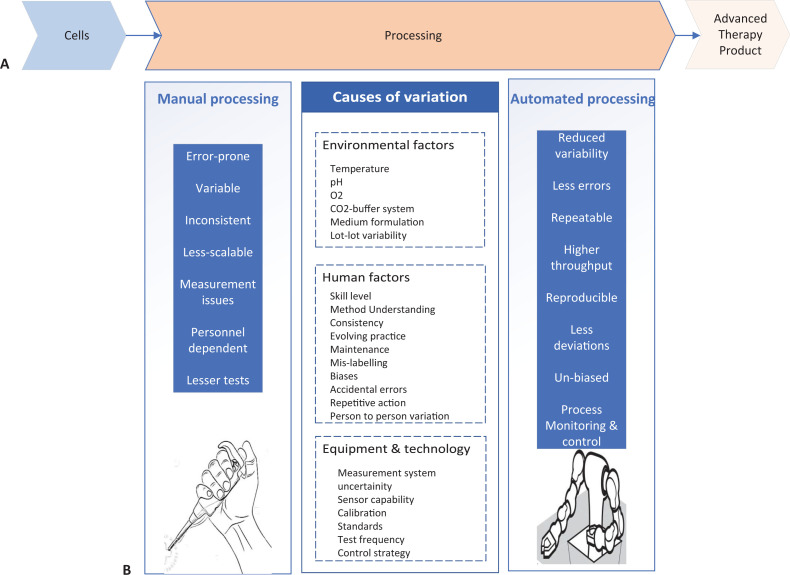
Comparison of manual and automated manufacturing of cell therapy products. (**A**) Schematic of the development lifecycle of cell therapy products. Cells (left) undergo processing in the laboratory (center), where they are cultured under different conditions, often requiring several complex cell culture steps, to develop safe and efficacious therapy product (right). (**B**) The processing step of therapy product development can be done manually (left) or automatically (right). Manual processing has several factors (center left) that increase variation in the culture conditions. Causes of variation can be environmental factors, human factors or equipment and technology factors (center), and they lead to manufacturing being less consistent. Automated processing can correct the issues that manual processing introduces (center right).

In recent years, advanced imaging techniques and analysis have improved the reproducibility of the cell culture protocols used for the manufacture of cells in regenerative medicine (Imai et al., 2018). This suggests that further minimizing human error and variability could also greatly improve the manufacturing process. One way to do this would be to mechanize and automate the cell culture procedures. Now, in eLife, Yosuke Ozawa, Koichi Takahashi, Tohru Natsume, and colleagues (who are based at various institutions in Japan) – with Genki Kanda and Taku Tsuzuki as first authors – report on LabDroid Maholo, a robotics and artificial intelligence-based cell processing station that can reliably carry out complex manual cell culture procedures typically performed by trained scientists (Kanda et al., 2022).

Replacing a difficult and repetitive manual process with a machine is a critical step in reducing the variability that arises in cell culture protocols. Indeed, it has previously been shown that automating methods using robotics is an efficient way to reduce human error and operator-dependent variability in the laboratory (Archibald et al., 2016). Kanda et al. now demonstrate this principle in the context of cell culture by combining robotics and artificial intelligence to automate complex cell culture protocols.

The LabDroid Maholo System contains highly accurate and programmable robotic arms to manipulate the cell culture conditions, which allowed the robot to culture stem cells under different conditions. In this case, the LabDroid Maholo System was programmed to differentiate stem cells into retinal pigment epithelial cells. The resulting cells were imaged using the LabDroid Maholo’s integrated imaging system, and computer algorithms and statistical analyses were applied to analyze and quantify the level of cell differentiation against the given conditions using the captured images.

The data obtained from these images was then fed into a mathematical experimental design algorithm that uses a batch Bayesian optimization. This allowed the LabDroid Maholo System to determine which factors in the cell culture procedure needed to be optimized to produce more retinal pigment epithelial cells. Using this method allowed Kanda et al. to reduce millions of potential combinations of cell culture parameters to a more manageable number. Briefly, the batch Bayesian optimization used by Kanda et al. first compares the cell culture conditions against the obtained rate of differentiation, and then decides, with the help of artificial intelligence, whether to include further optimization steps. In this case, three successive optimization steps successfully identified the best cell culture conditions to repeatably differentiate stem cells into retinal pigment cells of adequate quality to be used in cell therapy research.

Kanda et al. provide one of the best examples of how to achieve process control and advanced optimization using a combined robotics and artificial intelligence system. The ability to characterize the cultured cells and establish an optimal cell culture protocol based on the data removes human intervention for these steps, reducing variability. Although the platform will need further improvements to provide end-to-end functionality, it is a step forward towards the reproducible manufacturing of cells and cell-derived products for regenerative treatments.
